# Differences in Cortisol Response to Trauma Activation in Individuals with and without Comorbid PTSD and Depression

**DOI:** 10.3389/fpsyg.2017.00797

**Published:** 2017-05-18

**Authors:** Sharon Dekel, Tsachi Ein-Dor, Jeffrey B. Rosen, George A. Bonanno

**Affiliations:** ^1^Post-Traumatic Stress Disorder Program, Department of Psychiatry, Massachusetts General HospitalBoston, MA, USA; ^2^Department of Psychiatry, Harvard Medical SchoolBoston, MA, USA; ^3^School of Psychology, Interdisciplinary CenterHerzliya, Israel; ^4^Department of Psychology, University of DelawareNewark, NJ, United States; ^5^Department of Counseling and Clinical Psychology, Columbia UniveristyNew York, NY, United States

**Keywords:** PTSD symptoms, depressive symptom, September 11 terrorist attacks, cortisol, traumatic stress

## Abstract

**Background:** Although depression symptoms are often experienced by individuals who develop posttraumatic stress disorder (PTSD) following trauma exposure, little is know about the biological correlates associated with PTSD and depression co-morbidity vs. those associated with PTSD symptoms alone.

**Methods:** Here we examined salivary cortisol responses to trauma activation in a sample of 60 survivors of the World Trade Center attacks on September 11, 2001. Participants recalled the escape from the attacks 7 months post 9/11. Salivary cortisol levels were measured before and after their recollection of the trauma. PTSD, depression, and somatic symptoms were also assessed. From the behavioral assessment scales, the participants were grouped into three conditions: those with comorbid PTSD and depressive symptoms, PTSD alone symptoms, or no-pathology.

**Results:** Baseline and cortisol response levels differed between the comorbid, PTSD alone, and no-pathology groups. Individuals endorsing co-morbid symptoms had higher PTSD and somatic symptom severity and their cortisol response decreased following their trauma reminder while a trend of an elevated response to the trauma was found in the PTSD alone group. Our findings show distinct psychological and biological correlates related to the endorsement of PTSD with and without depression comorbidity.

**Conclusions:** The findings suggest that comorbidity symptoms manifestation entails a separate trauma induced condition from PTSD. Future research on biological correlates of comorbid PTSD and depression is warranted.

## Introduction

A significant number of individuals who encounter a traumatic event during the course of their lives experience temporary psychiatric symptoms, yet some go on and develop enduring and debilitating psychiatric conditions. Posttraumatic stress disorder (PTSD) is the most commonly associated disorder induced by trauma exposure (Breslau et al., 1997; Dekel et al., 2013b). Furthermore, many individuals endorsing PTSD report symptoms of depression as well (Kessler et al., 1995; O'Donnell et al., 2004), with comorbidity as high as 70% of the cases (Dekel et al., 2014). Considerable research has documented that the clinical expression of comorbid depression and PTSD differs from the presentation of PTSD alone (Sher, 2005). Comorbidity is associated with greater trauma exposure (Morina et al., 2013), PTSD symptom severity (Ikin et al., 2010), higher level of impairment (Mollica et al., 1999), sucidiality (Oquendo et al., 2003), and more hospitalizations (Sher, 2005). Depression may further mediate and intensify associated symptoms of PTSD such as somatization (Gupta, 2013) and contribute to an enduring sense of perceived threat (Lancaster et al., 2016). Although this clinical expression of the comorbidity condition suggests a more severe and enduring pathology, associated (unique) biological factors are relatively unknown. The present study explored potential differences in the biological stress response of individuals with PTSD and those endorsing PTSD as well as depressive symptoms.

The hypothalamic-pituitary adrenocortical (HPA) axis is an important part of the biological response to stress, promoting adaptation and accommodation to threatening and traumatic exposure (Gillespie et al., 2009). Stress exposure triggers a cascade of events in HPA activity with the end product being the release of the glucocorticoid hormone cortisol. Cortisol is generally elevated following trauma exposure (Kotozaki and Kawashima, 2012). In the short run cortisol facilitates the stress response and promotes the system's homeostasis and allostasis (Schulkin et al., 1994). However, prolonged activation of the HPA response can result in aggregated physiological and psychological wear and tear (McEwen, 2003). Cortisol abnormalities are associated with PTSD alone and depression alone (Morris et al., 2012b), but little is know about the cortisol pattern in comorbid PTSD and depression.

Neuroendocrine studies have consistently found cortisol variations in PTSD, which may in part account for the pathogenesis of the disorder. In general, cortisol (basal) abnormalities in PTSD have been largely manifested in hypoactivation of the system. Individuals with PTSD following a single traumatic event tend to have lower basal cortisol levels than healthy or trauma-exposed individuals without PTSD (Meewisse et al., 2007). The blunted cortisol response in PTSD is attributed to an enhanced HPA feedback function (Yehuda, 2002) leading to a progressive sensitization of the HPA-axis (Kendall-Tackett, 2000). Interestingly, depression is generally associated with hypercortisolemia (Holsboer, 2001), increased activity of the HPA axis and reduced inhibitory feedback (Kendall-Tackett, 2000). These findings raise the question whether individuals with PTSD + depression would differ from individuals with PTSD based on their HPA stress response.

Studies examined basal cortisol levels in individuals with co-morbid depression and PTSD reveal mixed findings. A few studies have documented that their (basal) cortisol levels are similar to those of healthy individuals, suggesting that the contrasting HPA patterns in depression and PTSD “counterbalance” one another when conditions co-occur (Halbreich et al., 1989). However, other recent studies have documented, lowered cortisol (basal) levels in the co-morbid group, as seen in pure PTSD, suggesting that comorbid depression does not effect the predominant PTSD cortisol pattern. For example, in a recent meta-analysis lower basal diurnal cortisol was evident in both PTSD as well as PTSD + depression groups relative to non-trauma exposed individuals (Morris et al., 2012a). Similarly, studies using pharmacological challenge tests [e.g., dexamethasone suppression test (DST)] for the most part conclude no effects of depression on HPA activity in PTSD (de Kloet et al., 2006). These results suggest that individuals with comorbid PTSD and depression resemble individuals with PTSD alone.

An important factor in understanding trauma-related disorders is the cortisol response to stressful stimulus and trauma-related reminders. Psychological distress and physiological reactivity upon exposure to trauma-related cues (DSM-5 B.4, B.5) are a salient character of PTSD. The failure of extinction of fear responsiveness to trauma-related cues is an underlying feature of the disorder. Responses to trauma stimulus provide a strong method for examining biological abnormalities in PTSD and co-morbid conditions. A consistent picture has emerged demonstrating greater peripheral nervous system reactivity including autonomic hyper-reactivity to trauma-related stimuli in individuals who develop PTSD (Dekel et al., 2016). Examining cortisol response in PTSD under symptom provocation may therefore offer a more promising assessment (Pitman et al., 2012) than measures of cortisol at rest.

In contrast to low basal levels, studies examining cortisol response in PTSD to stressful stimuli and trauma-reminders are few and for the most part point the direction of augmented cortisol responses (Liberzon et al., 1999; Bremner et al., 2003; Elzinga et al., 2003; Dekel et al., 2013a). Although these augmented cortisol responses are associated with PTSD, the contribution of comorbid depression symptoms remains unclear. Depression is associated with a sense of perceived threat (Lancaster et al., 2016) and may modify cortisol response to trauma reminder. Somatization symptoms are also frequently associated with depression and interact with the activity of the HPA-axis (Rief and Auer, 2000). To the best of our knowledge no study has compared the cortisol response to trauma-related reminders in comorbid depression + PTSD to that of PTSD alone. Thus, in the present study we examined cortisol response induced by trauma recollections in a sample of high-exposure 9/11 survivors. We initially examined differences in trauma exposure and symptom severity. We focused our analysis on the question whether individuals with comorbid PTSD and depressive symptoms differ from those who suffer from PTSD alone in their cortisol response.

## Methods

### Participants

The present analysis is part of a larger inquiry on psychological adaption to terrorism of Bonanno et al. (2005). Data at 7 months (Time 1: March, 2001, [T1]) and 18 months (Time 2: April, 2002, [T2]) post- 9/11 were obtained from individuals who had been in or within four blocks of the World Trade Center (WTC) towers at the time of the 9/11 attacks. Following approval from Columbia University's Institutional Review Board, we enlisted participants through their employers in the WTC, posted flyers in downtown Manhattan, and through public announcements. All participants signed written consent forms. The final prospective sample of individuals with data at the two assessment points included 60 participants (29 men and 31 women); the average age was 39.30 years (*SD* = 10.51), average annual income was $70,000, ethnically 77% were White (*n* = 48), and 44% resided in Manhattan (*n* = 28). There were no gender differences in demographics.

### Measures

*Threat exposure* during the event was measured by six items pertaining to feelings of physical danger and being highly distressed (“perceived danger”), assessed on a 0 = no to 4 = very much; scale (α = 0.83), and four items pertaining to witnessing deaths and injuries (“objective exposure”), assessed on a 0 = none to 3 = three or more; scale (α between 0.70 and 0.80). Exploratory factor analysis supported 2-factor structures for the measure at T1.

*PTSD symptoms* at T1 and T2 were measured using the PTSD Symptom Scale-Self Report (PSS-SR, Foa et al., 1993), a 17-item scale reflecting frequency and severity of PTSD symptoms according to the Diagnostic and Statistical Manual of Mental Disorders (American Psychiatric Association, 2013) in the past month using a 0 (never/rarely) to 3 (almost always) scale. The PSS-SR has produced adequate internal consistency (.91) and concurrent validity (Foa et al., 1993). Internal consistency in the present study was 0.91. Previous research indicates that a PSS-SR total score of 14 serves as a reliable cutoff score for screening for PTSD (Coffey et al., 2006).

*Depressive* symptoms at T1 and T2 were measured using the Center for Epidemiologic Studies Depression Scale, Brief Version (CES-D, Kohout et al., 1993), consisting of nine-item scale and has evidenced reliability and validity statistics comparable to the full-scale version (Kohout et al., 1993). Respondents used a 1 (hardly ever) to 5 (almost always) scale to indicate how often they experienced depression symptoms in the past 2 weeks. Internal consistency for the nine-item scale in the current study was 0.79. A CES-D total score of 16 is the standard cutoff for low vs. high depression symptoms.

*Somatic symptoms* were assessed at T1 with an 18-item self-report checklist used in the Whitehall II study, a large scale study of morbidity among civil servants in London, England (Marmot et al., 1991; Stansfeld et al., 1993).

*Trauma recollections* were obtained at T1 in a 30-min open-ended standardized interview asking participants to freely recall their personal experiences escaping from the attacks prompted by the instruction: “I would like you to speak about what you went through on September 11; your experiences, thoughts and feelings on that day” (Dekel and Bonanno, 2013). Participants were instructed to report as openly as possible about virtually anything that came to mind.

*Salivary cortisol* measured at T1 was collected 2 min before and after the 30-min interview using Salivette collection devices (Sarstedt, Newton, NC) and stored at −20°C. Salivary cortisol was analyzed by a high-sensitivity cortisol enzyme immunoassay (Salimetrics, State College, PA) as described in Dozier et al. (2006). Intra-assay (2.20%) and interassay (5.70%) variability and pH of the assays were within the range of precision expected by Salimetrics. Cortisol concentration (μg/dl) was measured by optical density of duplicate samples using a Dynex spectrophotometer.

## Results

First, we categorized participants as having a high or low number of symptoms of depression and PTSD. Categorization of a high vs. low number of depression symptoms was based on the standard cutoff for the CES-D Scale of 16; categorization of a high vs. low number of PTSD symptoms was based on the standard cutoff for PSS-SR Scale of 14. Next, we categorized participants into one of three psychopathology groups: “comorbid group,” i.e., high levels of both PTSD and depressive symptoms (*n* = 18); “PTSD group,” i.e., high levels of PTSD symptoms alone (*n* = 15); and “no-pathology group,” i.e., low levels of PTSD and depressive symptoms (*n* = 27). Only 3 participants had elevated depressive symptoms alone (without PTSD) and their data were not included in the analysis. There were no gender differences between study groups.

### Do the psychopathology groups differ in the extent of perceived danger and objective exposure to the WTC attacks?

To examine whether the study groups (comorbid, PTSD only, and no-pathology) differ in the extent of perceived danger and objective exposure to WTC attacks, we conducted two separate tests of one-way analysis of variance (ANOVA). Means, standard deviations, statistics and effect sizes are presented in Table 1.

**Table 1:** **Means, standard deviations, statistics, and effects sizes for the differences between psychopathology groups in perceived danger and objective exposure to 9/11 events**.

	**PTSD** + **Depression (*****N*** = **18)**		**PTSD only (*****N*** = **15)**		**No-pathology (*****N*** = **27)**			
	***M***	***SD***	***M***	***SD***	***M***	***SD***	***F*_(2, 56)_**	**η^2^**
Perceived danger	2.41^a^	1.30	2.18^a^	0.99	1.31^b^	0.98	6.26^**^	0.18
Objective exposure	1.06	0.79	0.88	0.81	0.67	0.83	1.25	0.04

** *p < 0.01. Means with different superscript are significantly different at p < 0.05 based on Sidak adjustment*.

The analyses indicated that the study groups significantly differed in the extent of perceived danger but not in the objective exposure to the 9/11 events. Sidak *post-hoc* analyses revealed that participants classified as comorbid PTSD + depressive symptoms or PTSD alone perceived the danger of the attacks to be higher than those of the no-pathology group (Šidák, 1967).

### Do the psychopathology groups differ in the severity of PTSD and somatic-related symptoms?

To examine whether the study groups (comorbid, PTSD only, and no-pathology) differ in the intensity of PTSD symptoms 7 and 18 months after the attacks and in somatic symptoms 7 months post the attack, we conducted multivariate analysis of variance (MANOVA). Means, standard deviations, statistics and effect sizes are presented in Table 2.

**Table 2:** **Means, standard deviations, statistics, and effects sizes for the differences between psychopathology groups in PTSD and somatic-related symptoms**.

	**PTSD** + **Depression (*****N*** = **18)**		**PTSD only (*****N*** = **15)**		**No-pathology (*****N*** = **27)**			
	***M***	***SD***	***M***	***SD***	***M***	***SD***	***F*_(2, 56)_**	**η^2^**
PTSD (7-months)	28.57^a^	7.62	19.69^b^	3.66	7.45^c^	4.82	61.47^***^	0.74
PTSD (18-months)	25.60^a^	9.36	13.36^b^	8.15	7.33^c^	5.44	24.41^***^	0.53
Somatic (7-months)	9.21^a^	2.91	6.15^b^	2.73	3.70^c^	2.20	18.87^***^	0.46

^**^p < 0.01. Means with different superscript are significantly different at p < 0.05 based on Sidak adjustment.

The analysis indicated that the study groups significantly differed in the multivariate factor of symptom severity, *Pillai's T* = 0.86, *F*_(6, 86)_ = 10.72, *p* < 0.001. Complementary one-way ANOVAs and discriminant analysis indicated that the comorbid group suffered from significantly higher intensity of PTSD symptoms (7 and 18 months) and somatic-related symptoms than the PTSD and no-pathology groups, with the largest differences in PTSD symptoms at 7 months (β = 0.94), followed by PTSD symptoms at 18 months (β = 0.58), and somatic-related symptoms at 7 months (β = 0.52). Both the comorbid and PTSD only groups had higher levels of PTSD symptom and somatic-related symptom severity than the no-pathology group.

### Do the psychopathology groups differ significantly in the cortisol reactivity to the WTC recollections?

First, we examined differences in baseline levels of cortisol between PTSD groups (PTSD + MDD, PTSD only, resilience), and we conducted one-way analysis of variance (ANOVA) with Brown-Forsythe adjustment to account for inequality of variance (a correction to the degrees of freedom). The analysis revealed significant results, *F*_(2, 25.96)_ = 3.85, *p* = 0.034, η^2^ = 0.16. Although the effect size was small, planned contrasts with Bonferroni correction because of non-orthogonal comparisons indicated that the baseline cortisol level of the PTSD + depression group (*M* = 0.17, *SD* = 0.12) was significantly higher than that of the PTSD only group (*M* = 0.10, *SD* = 0.06, *p* = 0.048) and/or that of the resilience group (*M* = 0.10, *SD* = 0.07, *p* = 0.020).

Next, to examine whether the study groups significantly differed in their cortisol reactivity, we conducted a mixed-designed analysis of covariance, in which the between-subject independent variable was study group (comorbid, PTSD only, no-pathology), the within-subject independent variable was time (before, after interview), and the dependent variable was level of cortisol. Because the groups differed in the extent of perceived danger, we adjusted the analysis for its contribution. The μg/dl of salivary cortisol means and standard deviations are presented in Table 3.

**Table 3:** **Means and standard deviations for the differences between psychopathology groups in cortisol level**.

	**PTSD + Depression (*N* = 18)**		**PTSD only (*N* = 15)**		**No-pathology (*N* = 27)**	
	***M***	***SD***	***M***	***SD***	***M***	***SD***
Cortisol level before	0.18	0.12	0.10	0.06	0.10	0.07
Cortisol level after	0.12	0.07	0.15	0.18	0.11	0.08

**Figure 1:**
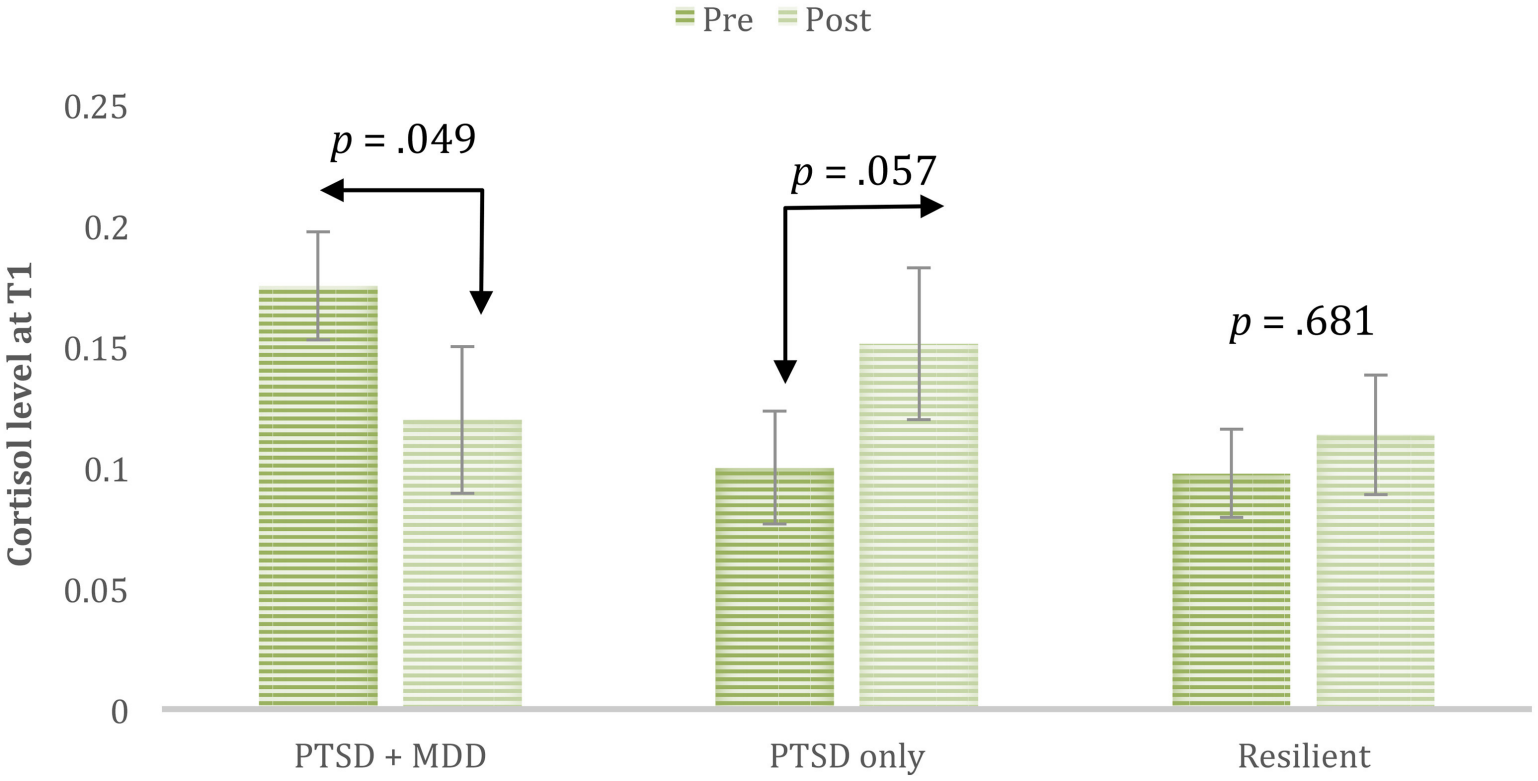
**Cortisol reactivity by psychopathology group**.

While there were no main effects of group or time, the analysis revealed a significant interaction between study group and time, *F*_(2, 43)_ = 4.22, *p* = 0.021, η^2^_*p*_ = 0.16. Simple effects tests with Sidak adjustment revealed distinct differences in cortisol reactivity between the psychopathology groups (see Figure 1). Whereas, the cortisol significantly decreased among the comorbid group, it marginally increased in the PTSD group (and remained the same among the no-pathology group).

## Discussion

There are two competing theories concerning the tight relationship between PTSD and depression associated with trauma exposure (Flory and Yehuda, 2015). First, the comorbidity manifestation reflects merely overlapping symptoms of the two conditions, (Southwick et al., 1991; Franklin and Zimmerman, 2001). Second, comorbid PTSD and depression represents a distinct trauma-induced phenotype (O'Donnell et al., 2004; Dekel et al., 2014). We explored predictions from these theories by measuring cortisol levels among high-exposure survivors of the WTC attacks in response to recalling their personal experience escaping from the attack 7 months after the event.

Our study is the first to show that individuals with comorbid PTSD and depressive symptoms have decreased cortisol reactivity following trauma reminder while those with PTSD symptoms alone and healthy trauma-exposed do not. A trend of an elevated rather than decreased response to the trauma reminder was found in the PTSD alone group. The data also reveal that individuals endorsing comorbid symptoms have higher PTSD symptoms and somatization than individuals with PTSD alone.

The findings of comorbid PTSD and depression. Ourelevated symptoms associated with the comorbid condition indicate that phenotypically this condition is more severe than PTSD alone, as previously noted (Sher, 2005). However, the cortisol data demonstrating differences in baseline and activation cortisol levels between the comorbid and the PTSD only groups may suggest distinct conditions and not only one of degree. Our findings are in accord with the few biological studies comparing functional and molecular processes, such as lower amygdala activation (Kemp et al., 2007) and lower epigenetic methylation (Yehuda et al., 2015) in PTSD alone vs. comorbid PTSD and depression. Our findings, along with these studies, demonstrate that individuals with comorbid symptoms differ biologically from those with PTSD; possibly they all or some suffer from separate trauma-related psychopathologies. Furthermore, the differential cortisol responses to retelling the trauma highlight the importance of trauma-activation, and not only basal cortisol levels, in accounting for depression associated with PTSD and PTSD alone psychopathologies.

Baseline levels of cortisol agree fairly well with the general views of cortisol in major depressive disorder and PTSD (Yehuda, 2002). That is, baseline cortisol levels of those with depressive symptomology had higher baseline levels than the PTSD only group. With the trauma reminder, those with depressive symptomology had a blunted cortisol response to trauma-activation, whereas the PTSD only group had a strong trend toward a sensitized cortisol response to trauma-activation. While both groups had PTSD symptoms, cortisol levels moved in different directions after trauma-activation suggesting that when depressive symptoms are comorbid with PTSD, the depressive HPA and cortisol mechanisms predominate over the PTSD mechanisms. Blunt cortisol associated with depressive symptoms may relate to mechanisms of psychological disengagement with environmental stressors (Burke et al., 2005) and excessive shutting down of the hypothalamic-pituitary-adrenal cortical (HPA) axis due to impairment in negative feedback. An enhanced cortisol response in PTSD alone may be related with re-experiencing symptoms (Dekel et al., 2013a). On the other hand, the comorbidity may have very different pathological mechanisms than either PTSD or depression alone. These speculations should be followed up in future research.

Several limitations in this study should be noted. We obtained only a single post-interview measure of cortisol, and we did not include information on menstrual cycle, oral contraceptive use, medications, smoking, eating, and physical exercise just before the interview. Although we measured PTSD and depressive symptoms, whether a participant had clinically diagnosed PTSD or depression was not asked. The small sample size, although commonly used in studies like ours, raises the possibility of sample bias, and in the case of baseline cortisol levels, a significant but small effect size. The study assessed cortisol levels and responses 7 months post 9/11 and we might have overlooked variations in the cortisol response associated with symptom manifestation in the more immediate aftermath of trauma exposure or in the recovery 18 months after 9/11.

## Conclusion

In summary, this study demonstrates higher baseline cortisol levels and lowered HPA-axis response correlates with PTSD + depressive symptoms and not with PTSD alone in individuals when confronted by trauma-reminders of the 9/11 WTC terrorist attacks. Importantly, trauma recollection, even 7 months post-trauma, might be an important tool to assess prolonged effects of trauma and their biological correlates. Biological markers of trauma-related psychopathology may offer an objective clinical assessment in addition to the existing DSM mental health classification based on subject description of symptoms. Further studies are needed to elucidate distinct biological abnormalities of various trauma-related conditions. A clearer understanding of unique biological targets may improve therapeutic interventions for trauma-exposed individuals endorsing comorbidity symptoms.

## Ethics statement

The study was approved by Teachers College, Columbia University Review Board. All subjects signed written consent.

## Author contributions

SD collected the study data, initiated and generated the study paradigm, and prepared and wrote the manuscript. TE conducted the main statistical analysis and contributed to the writing of the manuscript. JR conducted the cortisol analysis and contributed to the writing of the manuscript. GB is the principle investigator of 9/11 study, which the sample of the current study is derived from. GB contributed to the manuscript editing.

## Funding

This study was support by a Brain and Behavior Research Foundation (NARSAD) Young Investigator grant awarded to SD and her ECOR Claflin Distinguish Scholar award. GB received grants BCS-0202772 and BCS-0337643 from the National Science Foundation.

### Conflict of interest statement

The authors declare that the research was conducted in the absence of any commercial or financial relationships that could be construed as a potential conflict of interest.
